# Treatment of rats with *Jiangzhi* Capsule improves liquid fructose-induced fatty liver: modulation of hepatic expression of SREBP-1c and DGAT-2

**DOI:** 10.1186/s12967-015-0529-6

**Published:** 2015-06-02

**Authors:** Yuanyang Zhao, Yongquan Pan, Yifan Yang, Robert Batey, Jianwei Wang, Yuhao Li

**Affiliations:** Faculty of Basic Medical Sciences, Chongqing Medical University, Chongqing, China; The Laboratory Animal Center, Chongqing Medical University, Chongqing, China; Endocrinology and Metabolism Group, Sydney Institute of Health Sciences/Sydney Institute of Traditional Chinese Medicine, Sydney, NSW Australia; Central Clinical School, Royal Prince Alfred Hospital, The University of Sydney, Sydney, NSW Australia; Laboratory of Traditional Chinese Medicine, Chongqing Medical University, Chongqing, China

**Keywords:** Acyl-coenzyme A:diacylglycerol acyltransferase, Lipid, *Jiangzhi* Capsule, Liver, Sterol regulatory element-binding protein-1c, Triglyceride

## Abstract

**Background:**

*Jiangzhi* Capsule is an Australian listed patented traditional Chinese medicine and has been used for management of lipid abnormalities over the past 10 years. To obtain a better understanding regarding *Jiangzhi* Capsule, the present study investigated the effects and underlying mechanisms of *Jiangzhi* Capsule on chronic fructose overconsumption-induced lipid abnormalities.

**Methods:**

Male rats were treated with liquid fructose in their drinking water over 14 weeks. *Jiangzhi* Capsule was co-administered (once daily, by oral gavage) during the last 7 weeks. Indexes of lipid and glucose homeostasis were determined enzymatically, by ELISA and/or histologically. Gene expression was analyzed by real-time PCR, Western blot and/or immunohistochemistry.

**Results:**

Treatment with *Jiangzhi* Capsule (100 mg/kg) attenuated fructose-induced excessive triglyceride accumulation and Oil Red O-stained area in the liver. This effect was accompanied by amelioration of hyperinsulinemia. There was no significant difference in intakes of fructose and chow, and body weight between fructose control and fructose *Jiangzhi* Capsule-treated groups. Mechanistically, *Jiangzhi* Capsule downregulated fructose-stimulated hepatic overexpression of sterol regulatory element binding protein (SREBP)-1/1c at the mRNA and protein levels. Accordingly, the SREBP-1c downstream genes, acetyl-CoA carboxylase-1 and stearoyl-CoA desaturase-1, were also inhibited. In addition, acyl-coenzyme A:diacylglycerol acyltransferase (DGAT)-2 expression at the mRNA and protein levels in the liver was also inhibited after *Jiangzhi* Capsule treatment. In contrast, *Jiangzhi* Capsule affected neither carbohydrate response element binding protein, peroxisome proliferator-activated receptor (PPAR)-gamma and DGAT-1, nor PPAR-alpha and its target genes.

**Conclusions:**

These findings demonstrate the anti-steatotic action of *Jiangzhi* Capsule in fructose-fed rats, and modulation of hepatic SREBP-1c and DGAT-2 involved in hepatic de novo synthesis of fatty acids and triglyceride, respectively. Our findings provide an evidence-based and mechanistic understanding of *Jiangzhi* Capsule supporting its application for the prevention and/or treatment of fatty liver and its associated disorders in clinical practice.

**Electronic supplementary material:**

The online version of this article (doi:10.1186/s12967-015-0529-6) contains supplementary material, which is available to authorized users.

## Background

Nonalcoholic fatty liver disease has become an important public health problem due to its high prevalence, potential progression to severe liver disease, and association with cardiometabolic abnormalities [1, 2]. Fatty liver, the hallmark of nonalcoholic fatty liver disease, is linked to obesity, insulin resistance and type 2 diabetes [2]. However, there is no effective therapy currently approved by The Food and Drug Administration of the United States of America for treatment of this common disorder. Traditional Chinese medicine (TCM) has been used to treat liver disease in China since ancient times. Numerous Chinese herbs and active components have been tested for treatment of nonalcoholic fatty liver disease. Evidence from randomized controlled trials has suggested the efficacy and safety of TCM therapies in the treatment of nonalcoholic fatty liver disease [3]. TCM herbs have predominately been used clinically in the form of formulas containing an average of ten herbs [3]. However, relatively few evidence-based investigations have been undertaken to examine the therapeutic activities and the underlying mechanisms of action associated with TCM formulas for nonalcoholic fatty liver disease.

Excessive fat accumulation in the liver can occur as a result of numerous factors among which increased fat synthesis plays a pivotal role [2]. Hepatic de novo fatty acid synthesis may contribute to excessive lipid accumulation in the liver with the enzymes responsible for fatty acid synthesis being transcriptionally regulated [2]. Sterol regulatory element-binding protein (SREBP)-1c is the principal inducer of de novo hepatic lipogenesis by modulating lipogenic enzymes, such as acetyl-CoA carboxylase (ACC) and stearoyl-CoA desaturase (SCD)-1 [2, 4]. In contrast, acyl-coenzyme A:diacylglycerol acyltransferase (DGAT)s are the enzymes those catalyze the final step and rate-limiting reaction in triglyceride synthesis. DGAT-1 likely plays a role in intestinal repackaging of free fatty acids, whereas DGAT-2 is predominately expressed in the liver and catalyzes the final step of triglyceride biosynthesis [2]. Recent studies have demonstrated that DGAT-2 plays an important role in hepatocyte triglyceride synthesis, thereby contributing to hepatic steatosis [5, 6]. Reduction of DGAT-2 expression by antisense oligonucleotide attenuates hepatic steatosis in high fat diet-induced obese mice and *ob/ob* mice [7], and in high fat diet-fed rats [8].

*Jiangzhi* Capsule is an Australian listed patented TCM formula (AUST L 134445) and has been used for management of lipid abnormalities over the past 10 years. It is composed of 13 herbs: *Radix Astragali*, *Poria*, *Folium Nelumbinis*, *Rhizoma Alisma*, *FructusCrataegi*, *Fructus Chaenomelis*, *Radix et Rhizoma Salviae Miltiorrhizae*, *Radix et Rhizoma Notoginseng*, *Pollen Typhae*, *Rhizoma et Radix Polygoni cuspidati*, *Herba Taraxaci*, *Radix Polygoni multiflori* and *Fructus Ligustri Lucidi*. Many of individual herbs in this formula, such as *Rhizoma Alisma* [9], *Radix Salviae Miltiorrhizae* [10, 11], *Radix Notoginseng* [12–14] and *Fructus Ligustri Lucidi* [15] have been reported to regulate glucose and lipid metabolism and/or to protect the liver against injuries. Kwon et al. [16] found that the formula consisting of *Astragalus membranaceus*, *Crataegus pinnatiida*, *Alisma orientale*, *Salvia miltiorrhiza*, *Morus alba* and *Pueraria lobata* attenuated alcohol-induced fatty liver and liver damage in rats. Treatment with *FructusCrataegi* decreased hepatic SREBP-1c mRNA expression in apolipoprotein E-deficient mice [17]. Emodin, an active component contained in both *Radix Polygoni multiflori* and *Rhizoma et Radix Polygoni cuspidati* ameliorated high fat diet-induced excessive hepatic triglyceride accumulation, accompanied by a downregulation of hepatic SREBP-1c protein expression in rats [18]. We have recently demonstrated that oleanolic acid, one of the prominent active components contained in *Fructus Ligustri Lucidi*, improves fructose-induced fatty liver via the hepatic SREBP-1c pathway [19]. On the other hand, emodin has also been noted to decrease DGAT-1 content within in vitro models of steatosis hepatic L02 cell [20] in addition to reports highlighting water extracts of *Radix Polygoni multiflori* can decrease hepatic DGAT activity in high fat diet-fed rats [21]. Oleanolic acid has also been noted to inhibit DGAT activity in rat liver microsomes [22]. The tanshinones (cryptotanshinone, 15,16-dihydrotanshinone I, tanshinone IIA and tanshinone I) isolated from *Radix Salviae Miltiorrhiza* also showed inhibitory effect on DGAT activity in rat liver [23]. Whilst studies on the individual herbal components provide preliminary evidence, there is, however, a lack of evidence-based knowledge in the metabolic effects and the underlying mechanisms of *Jiangzhi* Capsule.

Fructose has now become a major constituent of our modern diet with chronic overconsumption increasing developmental risk of fatty liver, dyslipidemia, insulin resistance and obesity in animals and humans [4, 24]. Research has shown that sugar-sweetened nonalcoholic beverages, such as soft drinks, appear as the major source of fructose for all classes of age considered with the exception of children younger than 6 years and adults older than 50 years [4]. In the present study, we tested the effects of *Jiangzhi* Capsule on liquid fructose-induced lipid abnormalities and further investigated the underlying mechanisms in rats.

## Methods

### Preparation and identification of *Jiangzhi* Capsule

The raw herbs for *Jiangzhi* Capsule were purchased from Guangdong Yifang Pharmaceutical Co., Ltd, China, and identified by the botanist Dr. Dawen Zhao. The voucher specimens were deposited in Guangdong Yifang Pharmaceutical Co., Ltd, China. *Radix et Rhizoma Notoginseng* (*Panax notoginseng* (Burk.) F. H. Chen, voucher specimen no. S0020GZYJ106, 5%) was ground into find powder. *Radix Astragali* (*Astragalus membranaceus* (Fisch.) Bge. Var. *mongholicus* (Bge.) Hsiao, voucher specimen no. H0300/GZYJ118, 8%), *Poria* (*Poria cocos* (Schw.) Wolf, voucher specimen no. F0070/BJZY100, 8%), *Folium Nelumbinis* (*Nelumbo nucifera* Gaertn., voucher specimen no. H0100/GZYJ170, 3%), *Rhizoma Alisma* (*Alisma orientalis* (Sam.) Juzep., voucher specimen no. Z0030/BJZY083, 8%), *Fructus Crataegi* (*Crataegus pinnatiida* Bge. var. major N. E. Br., voucher specimen no. S0150/GZYJ173, 10%), *Fructus Chaenomelis* (*Chaenomeles speciosa* (Sweet) Nakai, voucher specimen no. M0160/GZYJ022, 6%), *Radix et Rhizoma Salviae miltiorrhizae* (*Salvia miltiorrhiza* Bge., voucher specimen no. D0100/BJZY032, 10%), *Pollen Typhae* (*Typha angustifolia* L., voucher specimen no. P0050/GZYJ182, 6%), *Rhizoma et Radix Polygoni cuspidati* (*Polygonum Cuspidatum* Sieb. et Zucc., voucher specimen no. H0113/BJZY135, 10%), *Herba Taraxaci* (*Taraxacum mongolicum* Hand.-Mazz., voucher specimen no. P0040/GZYJ142, 10%), *Radix Polygoni multiflori* (*Polygortum multiflorum* Thunb., voucher specimen no. H0360/BJZY063, 8%) and *Fructus Ligustri lucidi* (*Ligustrum lucidum* Ait., voucher specimen no. N0080/GZYJ138, 8%) were ground into crude powder and extracted with water for two times (10 volumes of water for 2 h boiling and 7 volumes of water for 1 h boiling). The combined filtrate was evaporated under reduced pressure below 50°C. The yield of the extract was 28%. The powdered *Radix et Rhizoma Notoginseng* and the extract were completely mixed to produce *Jiangzhi* Capsule used in the present study. For quality control, *Jiangzhi* Capsule was identified by HPLC process similar to that described in the Chinese Pharmacopoeia (Version 1, 2010). Briefly, HPLC profiles were performed on an Agilent 1100 ZG-0090 HPLC instrument with Agilent Chimstation System. The chromatography was carried out on an Agilent XDB-C_18_ 5 μm 250 × 4.6 mm (for determination of ginsenoside Rg1, ginsenoside Rb1 and notoginsenoside R1) or Purospher-star 5 μm 150 × 4.6 mm (for salvianolic acid B determination) column. The sample injection volume was 10 µl. The mobile phase for salvianolic acid B determination was consist of methanol, acetonitrile, formic acid and water (ratio of 30:10:1:59 respectively), while determination of ginsenoside Rg1, ginsenoside Rb1 and notoginsenoside R1 was a gradient consisting of a mixture of water and acetonitrile (0–40 min, 80:20; 40–50 min, 80 → 70:20 → 30; 50–74 min, 70:30; 74–84 min, 70 → 20:30 → 80; 84–100 min, 20:80). Pure salvianolic acid B, ginsenoside Rg1, ginsenoside Rb1 and notoginsenoside R1 (purchased from National Institutes for Food and Drug Control, Beijing, China) were used as external standards. Peak areas were quantified at 286 nm for salvianolic acid B, and at 203 nm for ginsenoside Rg1, ginsenoside Rb1 and notoginsenoside R1.

### Animals and treatment protocols

All animal procedures were conducted according to international, national and institutional rules regarding animal experimentation, and approved by the Animal Ethics Committee, Chongqing Medical University, China.

Male Sprague–Dawley rats weighing 210–230 g and the standard chow were supplied by the Laboratory Animal Center, Chongqing Medical University, China. Rats were housed in a temperature controlled facility (21 ± 1°C, 55 ± 5% relative humidity) with a 12-h light/dark cycle. Animals were allowed free access to water and the standard chow for at least 1 week prior to starting the experiments.

Fructose in drinking water used for the present study has been described previously [19, 25–28]. Thirty-three rats were divided initially into two groups: water control free access to water (n = 6), and fructose group free access to 10% fructose solution (w/v, preparation every day) (n = 27). This fructose group had continued free access to 10% fructose solution for the duration of the study but was further divided into the following three groups (n = 9) 7 weeks after study commencement: fructose control, fructose *Jiangzhi* Capsule 25 mg/kg and fructose *Jiangzhi* Capsule 100 mg/kg. Animals in *Jiangzhi* Capsule-treated groups were administered *Jiangzhi* Capsule 25 or 100 mg/kg (suspended in 5% Gum Arabic solution, gavage once daily), respectively. The rats in the corresponding water- and fructose-control groups received vehicle (5% Gum Arabic) alone. All rats had free access to the standard chow. To avoid stress and increase monitoring accuracy of fructose intake, only three rats were housed in a cage at any given time. The consumed chow and fructose solution were measured per three rats daily and the intake of fructose was calculated. At the endpoint of the experiment, rats were deprived of chow and fructose solution, but still had free access to water overnight. Blood samples were collected by retroorbital venous puncture under ether anesthesia at 9:00–12:00 am for determination of plasma concentrations of total cholesterol (kit from Kexin Institute of Biotechnology, Shanghai, China), triglyceride (Triglyceride-E kit, Wako, Osaka, Japan), non-esterified fatty acid (NEFA) (NEFA-C kit, Wako, Osaka, Japan), glucose (kit from Kexin Institute of Biotechnology, Shanghai, China) and insulin (kit from Morinaga Biochemical Industries, Tokyo, Japan). Animals were immediately weighed and euthanised by prompt dislocation of the neck vertebra. The liver was collected and weighed, and the ratio of liver weight to body weight calculated. Segments of liver were snap frozen in liquid nitrogen and stored at −80°C for subsequent determination of gene/protein expression, and triglyceride and total cholesterol contents.

### Determination of triglyceride and total cholesterol contents in liver

Triglyceride and total cholesterol contents in liver were determined as described previously [29]. Briefly, 100 mg of tissue was homogenized and extracted with 2 ml of isopropanol. After centrifugation (3,000 rpm), the triglyceride and total cholesterol contents in supernatants were determined enzymatically (Wako, Osaka, Japan).

### Histological examination

A portion of liver was fixed with 10% formalin and embedded in paraffin. Three-micron sections were cut and stained with hematoxylin and eosin for examination of liver histology (BX-51, Olympus Corporation, Tokyo, Japan). To further confirm lipid droplet accumulation, 6-μm frozen sections were stained with Oil Red O. Forty fields in three individual sections were randomly selected, and the Oil Red O-stained area and the total tissue area were measured using an ImageJ 1.43 analyzing system. The ratio of the Oil Red O-stained area to the total tissue area was calculated (%).

### Real-time PCR

Real time PCR was performed as described previously [25, 26]. Total RNA was isolated from the livers of individual rats using TRIzol (Takara, Dalian, China). cDNA was synthesized using M-MLV RTase cDNA Synthesis Kit (Takara, Dalian, China) according to the manufacturer’s instructions. Real time PCR was performed with the CFX 96 Real Time PCR Detection System (Biorad Laboratories Inc, Hercules, CA, USA) using the SYBR® Premix Ex Taq™ II (Takara, Dalian, China). The sequences of primers are shown in Additional file 1: Table S1. The gene expression from each sample was analysed in duplicates and normalized against the internal control gene β-actin. Levels in water control rats were arbitrarily assigned a value of 1.

### Western blot

Western blot was performed as described previously [25]. Total and nuclear proteins were prepared individually from livers using the kits for tissue and nuclear protein extraction (Pierce Biotechnology, Rockford, IL, USA), according to the manufacturer’s instructions. Protein concentration was determined using the Bradford method (Bio Rad Laboratories, Hercules, CA, USA) using bovine serum albumin as a standard. Protein (30 μg) was subjected to SDS-PAGE analysis on a 10% gel, and then electrotransferred onto polyvinylidene fluoride membrane (Amersham, Buckinghamshire, UK). SREBP-1 and DGAT-2 (dilution 1:200, Santa Cruz Biotechnology, Santa Cruz, CA, USA) were detected with a goat polyclonal antibody and rabbit polyclonal antibody, respectively. Detection of signals was performed using the ECL Western blot detection kit (Pierce Biotechnology, Rockford, IL, USA) with anti-goat and anti-rabbit horseradish peroxidase-conjugated IgG (dilution 1:5,000, Santa Cruz Biotechnology, Santa Cruz, CA, USA) as second antibody, respectively. Polyclonal rabbit Lamin A/C antibody (dilution 1:1,000, Cell Signaling Technologies, Beverly, MA, USA) was used as loading control to normalize the signal obtained for nuclear SREBP-1 protein. Mouse monoclonal β-actin antibody (dilution 1:1,000, Santa Cruz Biotechnology, Santa Cruz, CA, USA) was used as loading control to normalize the signal obtained for DGAT-2 protein. The immunoreactive bands were visualized by autoradiography and the density was evaluated using ImageJ 1.43. Levels in water control rats were arbitrarily assigned a value of 1.

### Immunohistochemistry

A portion of liver was fixed with 4% paraformaldehyde, dehydrated and embedded in paraffin. Sections (3 μm) were dewaxed in xylene, rehydrated in ethanol and treated with 3% H_2_O_2_ in absolute methanol for 30 min. Next, sections were immersed in citrate buffer (pH = 6.0), boiled for 10 min and cooled down at room temperature. Slides were blocked with normal goat serum for 30 min and then incubated with rabbit polyclonal anti-DGAT2 antibodies (dilution 1:200, Santa Cruz Biotechnology, Santa Cruz, CA, USA) at 4°C overnight. Next, the samples were submitted to ABC (kit from Zhongshan Golden Bridge Biotechnology, Beijing, China) (biotin: 1 h; streptavidin: 30 min, 37°C), followed by incubation with DAB (kit from Zhongshan Golden Bridge Biotechnology, Beijing, China) for 1 min. Counterstaining was performed with Mayer’s hematoxylin. Omission of the primary antibody was served as the negative control.

### Data analysis

All results are expressed as means ± SEM. Data were analyzed by ANOVA using the StatView software, and followed by The Student–Newman–Keuls test to locate the differences between groups. *P* < 0.05 was considered to be statistically significant.

## Results

### Identification of the contents of some active components in *Jiangzhi* Capsule

It has been reported that *Radix Salviae miltiorrhizae* [10, 11, 30] and *Radix Notoginseng* [12–14] have pleiotropic pharmacological activities. In the present study, some typical active components contained in *Radix Salviae miltiorrhizae* and *Radix Notoginseng* of *Jiangzhi* Capsule were identified and quantified by HPLC as follows: salvianolic acid B, 0.533% (retention time 10.755 min), ginsenoside Rg1, 0.787% (retention time 37.289 min), ginsenoside Rb1, 0.640% (retention time 71.332 min) and notoginsenoside R1, 0.199% (retention time 24.869 min).

### Intakes of fructose and chow, and body weight in rats

**Figure 1:**
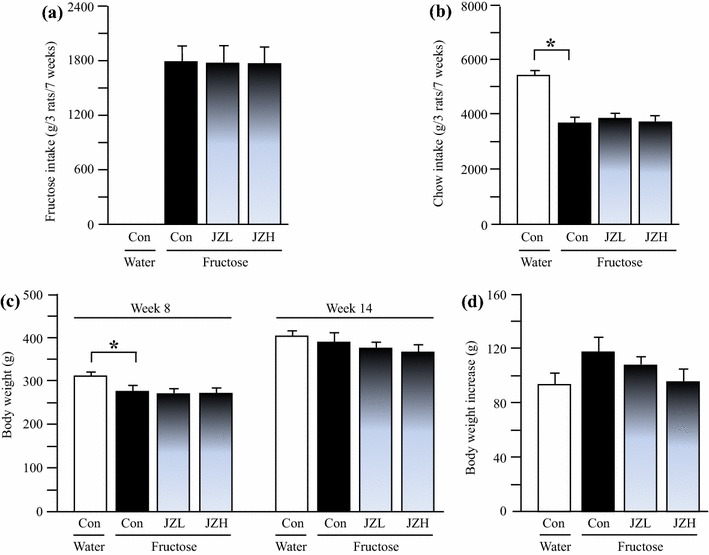
Intakes of fructose (**a**) and laboratory chow (**b**), body weight (**c**) and body weight gain (**d**) in water control, fructose control and fructose *Jiangzhi* Capsule (JZ)-treated rats. Data are means ± SEM (n = 6–9 each group). *P < 0.05. *Con* control, *JZL* JZ 25 mg/kg, *JZH* JZ 100 mg/kg.

The results showed that fructose control rats ate less chow, compared to water controls (Figure 1b). There was no significant difference in intakes of fructose (Figure 1a) and chow (Figure 1b) between fructose control and fructose *Jiangzhi* Capsule-treated groups. Water control rats were heavier than fructose controls; there was no difference in body weight between fructose control and fructose *Jiangzhi* Capsule groups before treatments commenced (Figure 1c). There was no difference in body weights between groups at the endpoint of the experiment (Figure 1c); the body weight gain in fructose control intended to increase compared to water control, which tended to decrease after *Jiangzhi* Capsule treatment (Figure 1d).

### Blood biochemical parameters in rats

**Figure 2:**
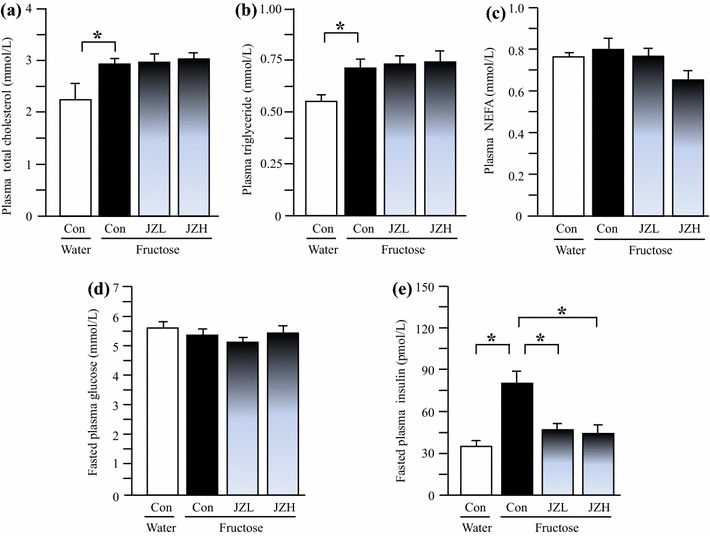
Plasma total cholesterol (**a**), triglyceride (**b**), NEFA (**c**), glucose (**d**) and insulin (**e**) concentrations in water control, fructose control and fructose *Jiangzhi* Capsule (JZ)-treated rats. Data are means ± SEM (n = 6–9 each group). *P < 0.05. *Con* control, *JZL* JZ 25 mg/kg, *JZH* JZ 100 mg/kg.

Compared to water control rats, fructose controls showed higher plasma concentrations of total cholesterol (Figure 2a), triglyceride (Figure 2b) and insulin (Figure 2e), whereas there was no significant difference in plasma NEFA (Figure 2c) and glucose (Figure 2d) concentrations between water control and fructose control. *Jiangzhi* Capsule at both 25 and 100 mg/kg significantly suppressed the insulin increase (Figure 2e). However, *Jiangzhi* Capsule showed minimal effect on plasma concentrations of total cholesterol, triglyceride, NEFA and glucose (Figure 2a–d).

### Liver-associated parameters in rats

**Figure 3:**
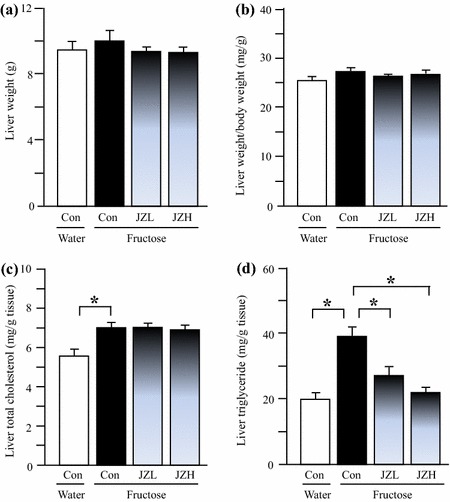
Liver weight (**a**), the ratio of liver weight to body weight (**b**), liver total cholesterol content (**c**) and liver triglyceride content (**d**) in water control, fructose control and fructose *Jiangzhi* Capsule (JZ)-treated rats. Data are means ± SEM (n = 6–9 each group). *P < 0.05. *Con* control, *JZL* JZ 25 mg/kg, *JZH* JZ 100 mg/kg.

**Figure 4:**
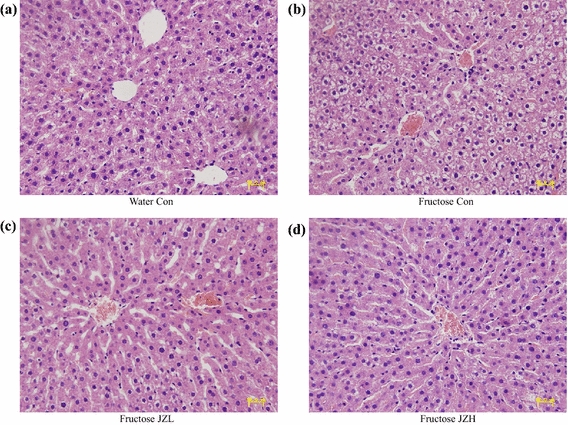
Representative images showing histology of liver (hematoxylin and eosin-staining, **a**–**d**, ×200) in water control, fructose control and fructose *Jiangzhi* Capsule (JZ)-treated rats. *Con* ontrol, *JZL* JZ 25 mg/kg, *JZH* JZ 100 mg/kg.

**Figure 5:**
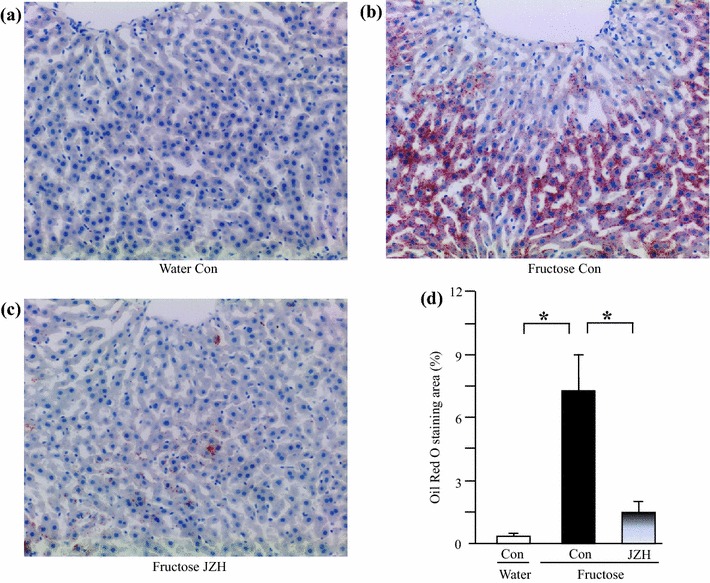
Representative images showing hepatic lipid accumulation (Oil Red O staining, **a**–**c**, ×200) and Oil Red O stained area (**d**) in water control, fructose control and fructose *Jiangzhi* Capsule (JZ)-treated rats. Data are means ± SEM (n = 6–9 each group). *P < 0.05. *Con* control, *JZH* JZ 100 mg/kg.

Although fructose feeding did not significantly affect liver weight (Figure 3a) and the ratio of liver weight to body weight (Figure 3b), hepatic total cholesterol (Figure 3c) and triglyceride (Figure 3d) contents were increased after fructose feeding. Accordingly, fructose feeding increased vacuolization (Figure 4b) and Oil Red O staining area (Figure 5b), indicative of fructose-induced excess hepatic lipid droplet accumulation. *Jiangzhi* Capsule treatment (both dosages) did not alter liver weight (Figure 3a), ratio of liver weight to body weight (Figure 3b) or hepatic total cholesterol content (Figure 3c). However, *Jiangzhi* Capsule substantially decreased hepatic triglyceride content (Figure 3d). This coincided with vacuolization (Figure 4c, d) and Oil Red O staining area (Figure 5c, d) in the liver being also significantly reduced.

### Hepatic gene/protein expression in rats

As the treatment with *Jiangzhi* Capsule at 100 mg/kg showed more pronounced effects on hepatic triglyceride accumulation, comparisons in gene/protein expression are restricted to water control, fructose control, and fructose *Jiangzhi* Capsule 100 mg/kg groups.

**Figure 6:**
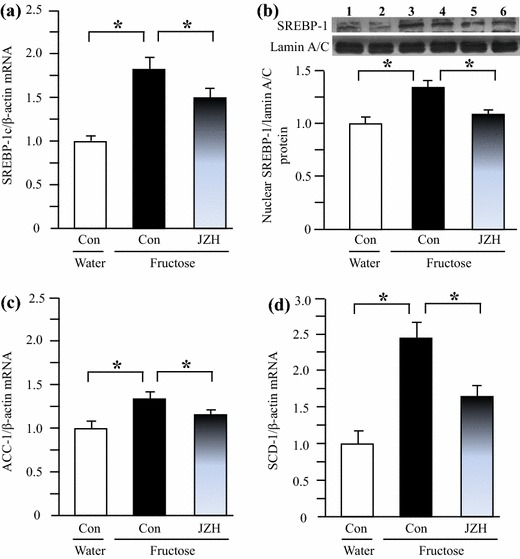
Hepatic expression of mRNAs encoding sterol regulatory element-binding protein (SREBP)-1c (**a**), acetyl-CoA carboxylase (ACC)-1 (**c**) and stearoyl-CoA desaturase (SCD)-1 (**d**), and protein of nuclear SREBP-1 (**b** lanes *1*, *2* water control; lanes *3*, *4* fructose control; lanes *5*, *6* fructose JZ 100 mg/kg) in water control, fructose control and fructose *Jiangzhi* Capsule (JZ)-treated rats. Data are means ± SEM (n = 6–9 each group). *P < 0.05. *Con* control, *JZH* JZ 100 mg/kg.

**Figure 7:**
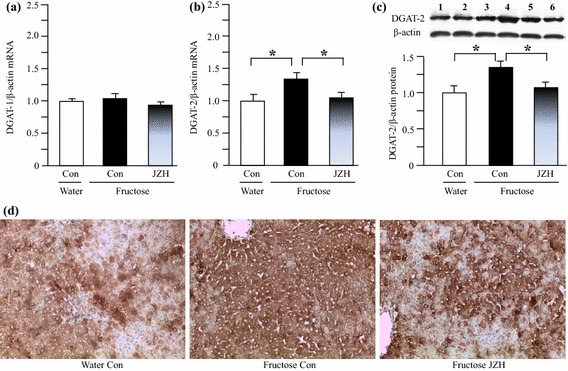
Hepatic expression of mRNAs encoding acyl-coenzyme A:diacylglycerol acyltransferase (DGAT)-1 (**a**) and DGAT-2 (**b**), and protein of DGAT-2 by Western blot (**c** lanes *1*, *2* water control; lanes *3*, *4* fructose control; lanes *5*, *6* fructose JZ 100 mg/kg) and immunohistochemical staining (**d**) in water control, fructose control and fructose *Jiangzhi* Capsule (JZ)-treated rats. Data are means ± SEM (n = 6–9 each group). *P < 0.05. *Con* control, *JZH* JZ 100 mg/kg.

By real-time PCR 14-week fructose feeding increased hepatic expression of mRNAs encoding SREBP-1c (Figure 6a), ACC-1 (Figure 6c), SCD-1 (Figure 6d) and DGAT-2 (Figure 7b). The increased contents of nuclear SREBP-1 protein (Figure 6b) and DGAT-2 protein (Figure 7c) were further demonstrated by Western blot analysis. Immunohistochemical staining results also showed upregulated DGAT-2 protein expression in fructose control compared to water control (Figure 7d). Seven-week *Jiangzhi* Capsule treatment downregulated mRNA levels of SREBP-1c (Figure 6a), ACC-1 (Figure 6c), SCD-1 (Figure 6d) and DGAT-2 (Figure 7b). The results of protein expression further confirmed the suppression of SREBP-1 (Figure 6b) and DGAT-2 (Figure 7c, d) by *Jiangzhi* Capsule treatment.

**Figure 8:**
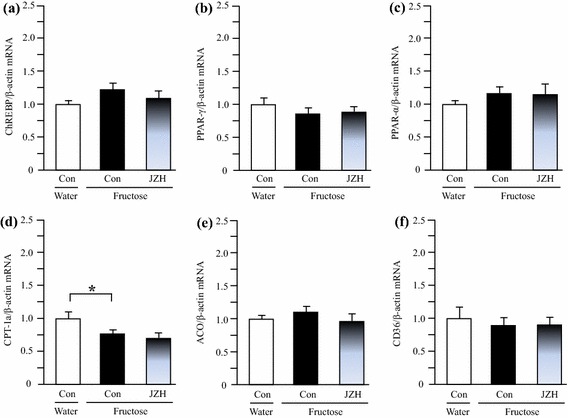
Hepatic expression of mRNAs encoding carbohydrate response element binding protein (ChREBP) (**a**), peroxisome proliferator-activated receptor (PPAR)-γ (**b**), PPAR-α (**c**), carnitine palmitoyltransferase (CPT)-1a (**d**), acyl-CoA oxidase (ACO) (**e**) and CD36 (**f**) in water control, fructose control and fructose *Jiangzhi* Capsule (JZ)-treated rats. Data are means ± SEM (n = 6–9 each group). *P < 0.05. *Con* control, *JZH* JZ 100 mg/kg.

Also with regards to the liver, fructose feeding did not significantly alter mRNA levels of DGAT-1 (Figure 7a), ChREBP (Figure 8a), PPAR-γ (Figure 8b), PPAR-α (Figure 8c), acyl-CoA oxidase (ACO) (Figure 8e) and CD36 (Figure 8f), but downregulated carnitine palmitoyltransferase (CPT)-1a expression (Figure 8d). *Jiangzhi* Capsule treatment showed minimal effect on expression of these genes (Figure 8a–f).

## Discussion

The present results clearly demonstrated that treatment of rats with *Jiangzhi* Capsule decreased fructose feeding-induced excess hepatic triglyceride accumulation and increased vacuolization and Oil Red O staining area in the livers. However, *Jiangzhi* Capsule did not affect chow and fructose intakes and body weight, and had minimal effect on plasma concentrations of total cholesterol, triglyceride, NEFA and glucose. Therefore, these findings suggest a specific anti-steatotic effect of *Jiangzhi* Capsule in rats.

Fructose, by providing large amounts of hepatic triose-phosphate as precursors for fatty acid synthesis, is highly lipogenic [4]. Recent findings suggest that the increase in hepatic de novo lipogenesis is one of the major providers of lipids in fructose-induced fatty liver [4, 31]. A high-fructose diet has been shown to induce the expression of the transcription factor SREBP-1c [4]. In addition, fructose consumption may also activate another hepatic transcription factor ChREBP, which upregulates the expression of hepatic lipogenic genes responsible for fatty acid synthesis [4]. In the present study, *Jiangzhi* Capsule treatment substantially suppressed fructose-stimulated hepatic overexpression of both SREBP-1c mRNA and nuclear SREBP-1 protein. Accordingly, the overexpression of SREBP-1c downstream genes ACC-1 and SCD-1 was also downregulated, however, hepatic ChREBP expression remained unchanged after *Jiangzhi* Capsule treatment. Thus, these results suggest that modulation of hepatic SREBP-1c-mediated expression of the genes responsible for hepatic de novo fatty acid synthesis contributes to the anti-steatotic effect of *Jiangzhi* Capsule in rats.

Recently, we have demonstrated that mangiferin, a prominent component contained in many anti-obese and anti-diabetic herbs, ameliorates fructose-induced fatty liver by suppressing hepatic DGAT-2 expression in spontaneously hypertensive rats [28]. In the present study, treatment with *Jiangzhi* Capsule also significantly inhibited hepatic DGAT-2 expression at the mRNA and protein levels, but was without effect on DGAT-1 expression in fructose-fed rats. Thus, these findings suggest that inhibition of hepatic DGAT-2 is also responsible for *Jiangzhi* Capsule-elicited attenuation of fructose-induced excessive hepatic triglyceride accumulation.

Reduction in hepatic fatty acid oxidation and increased fatty acid uptake into liver appear to have only minor roles in hepatic triglyceride deposition [2]. PPAR-α, predominantly expressed in the liver and, to a lesser extent, in the heart and muscle, has a crucial role in controlling fatty acid oxidation and uptake through direct transcriptional control of the genes, such as CPT1a, ACO and CD36 [32]. The induction of fatty acid oxidation by PPAR-α activation improves plasma lipid profiles. In a variety of mouse models, PPAR-α agonists lower plasma triglycerides, reduce adiposity and improve hepatic and muscular steatosis [32]. In contrast, PPAR-γ is predominantly expressed in adipose tissue and normally at low level in liver [32]. PPAR-γ is associated with regulation of the genes encoding molecules that promote a combination of lipid storage and lipogenesis [32]. In mice, activation of PPAR-γ in liver appears to contribute to the development of hepatic steatosis [33, 34]. It has been reported that the contribution of de novo lipogenesis to fructose-induced hypertriglyceridemia is small [35]. Furthermore, research findings suggest a DGAT-2-induced disconnection between liver and circulating triglyceride levels. In transgenic mice overexpressing hepatic DGAT-2, there were increased liver triglyceride content and reduced circulating triglyceride level [36, 37]. In the present study, treatment with *Jiangzhi* Capsule did not alter hepatic expression of PPAR-α, CPT-1a, ACO, CD36 and PPAR-γ in fructose-fed rats. Thus, our findings in gene expression do not support the involvement of the hepatic PPAR-α and PPAR-γ pathways in the anti-steatotic effect of *Jiangzhi* Capsule in rats. A consideration may be that modulation of hepatic de novo lipogenesis via the SREBP-1c and DGAT-2 pathways by *Jiangzhi* Capsule is insufficient to improve fructose-induced hypertriglyceridemia.

Although hepatic steatosis is strongly associated with the development of insulin resistance, it remains unclear whether insulin resistance causes the excessive accumulation of triglyceride in the liver, or whether the increase in triglyceride itself or of metabolite intermediates may play a causal role in the development of insulin resistance [2]. Some studies have shown that the accumulation of intrahepatic lipids precedes the state of insulin resistance [2]. In the present study, the anti-steatotic effect of *Jiangzhi* Capsule treatment was accompanied by pronounced amelioration of fructose-induced hyperinsulinemia. Further investigations are needed to determine whether *Jiangzhi* Capsule improves insulin resistance through attenuation of excessive hepatic triglyceride accumulation or whether the improvement of fatty liver is partially secondary to the amelioration of insulin resistance.

## Conclusions

Our present results demonstrate the anti-steatotic action of *Jiangzhi* Capsule in fructose-fed rats and modulation of hepatic SREBP-1c and DGAT-2 that are involved in hepatic de novo synthesis of fatty acids and triglyceride, respectively. Our findings provide an evidence-based and mechanistic understanding of *Jiangzhi* Capsule for the prevention and/or treatment of fatty liver and its associated disorders in clinic.

## Additional files

Additional file 1: Table S1. Primer sequences for real time PCR assays.

## Abbreviations

ACC: acetyl-CoA carboxylase
ACO: acyl-CoA oxidase
ChREBP: carbohydrate response element binding protein
CPT: carnitine palmitoyltransferase
DGAT: acyl-coenzyme A:diacylglycerol acyltransferase
NEFA: non-esterified fatty acids
PPAR: peroxisome proliferator-activated receptor
SCD: stearoyl-CoA desaturase
SREBP: sterol regulatory element-binding protein
TCM: traditional Chinese medicine
