# Supplementary material for: Treatment of rats with Jiangzhi Capsule improves liquid fructose-induced fatty liver: modulation of hepatic expression of SREBP-1c and DGAT-2
Source: J Transl Med. 2015 Jun 2;13:174. doi: 10.1186/s12967-015-0529-6 (PMC4467629; doi:10.1186/s12967-015-0529-6)
Supplement: Additional file 1: Table S1. — Primer sequences for real time PCR assays. [file 12967_2015_529_MOESM1_ESM.doc]

**Table 1. Primer sequences for Real Time PCR assays**

| Gene | Accession number | Primer Sequences |
| --- | --- | --- |
| β-actin | NM_031144.2 | Forward: ACGGTCAGGTCATCACTATCG |
|  |  | Reverse: GGCATAGAGGTCTTTACGGATG |
| ACC-1 | NM_022193.1 | Forward: AACATCCCGCACCTTCTTCTAC |
|  |  | Reverse: CTTCCACAAACCAGCGTCTC |
| ACO | NM_017340.2 | Forward: CCCAAGACCCAAGAGTTCATTC |
|  |  | Reverse: TCACGGATAGGGACAACAAAGG |
| CD36 | NM_001109218 | Forward: AACCCAGAGGAAGTGGCAAAG |
|  |  | Reverse: GACAGTGAAGGCTCAAAGATGG |
| ChREBP | FN432819.1 | Forward: GAAGACCCAAAGACCAAGATGC |
|  |  | Reverse: TCTGACAACAAAGCAGGAGGTG |
| CPT-1a | NM_031559.2 | Forward: CTGCTGTATCGTCGCACATTAG |
|  |  | Reverse: GTTGGATGGTGTCTGTCTCTTCC |
| DGAT-1 | NM_053437.1 | Forward: GGACAAAGACCGGCAGACCA |
|  |  | Reverse: CAGCATCACCACGCACCAAT |
| DGAT-2 | NM_001012345.1 | Forward: CCTGGCAAGAACGCAGTCAC |
|  |  | Reverse: GAGCCCTCCTCAAAGATCACC |
| PPAR-γ | AB_011365.1 | Forward: GCCCTTTGGTGACTTTATGGAG |
|  |  | Reverse: GCAGCAGGTTGTCTTGGATGT |
| PPAR-α | HM117640.1 | Forward: GTCATCACAGACACCCTCTCCC |
|  |  | Reverse: TGTCCCCACATATTCGACACTC |
| SCD-1 | NM_139192.2 | Forward: CAGTTCCTACACGACCACCACTA |
|  |  | Reverse: GGACGGATGTCTTCTTCCAGAT |
| SREBP-1c | XM_213329.5 | Forward: CTGTCGTCTACCATAAGCTGCAC |
|  |  | Reverse: ATAGCATCTCCTGCACACTCAGC |

Sequences: 5’ to 3’.
